# Di-μ-nitrito-κ^4^ *O*:*O*-bis­[bis­(1-ethyl-1*H*-imidazole-κ*N* ^3^)(nitrito-κ*O*)copper(II)]

**DOI:** 10.1107/S1600536811020745

**Published:** 2011-06-11

**Authors:** Run-Qiang Zhu

**Affiliations:** a Ordered Matter Science Research Center, College of Chemistry and Chemical Engineering, Southeast University, Nanjing 211189, People’s Republic of China

## Abstract

In the structure of the title compound, [Cu_2_(NO_2_)_4_(C_5_H_8_N_2_)_4_], the asymmetric unit consists of two moieties containing one Cu ion, two nitrite ions and two 1-ethyl-1*H*-imidazole mol­ecules associated *via* weak Cu—O inter­actions. Each Cu^II^ atom displays an elongted square-pyramidal CuN_2_O_3_ coordination geometry with a slight tetra­hedral distortion in the basal plane. The dimeric units are linked into a three-dimensional network by C—H⋯O hydrogen bonds.

## Related literature

For general background on ferroelectric metal–organic compounds with framework structures, see: Fu *et al.* (2009 ▶); Ye *et al.* (2006 ▶); Zhang *et al.* (2008 ▶, 2010 ▶). For a related structure, see: Costes *et al.* (1995 ▶).
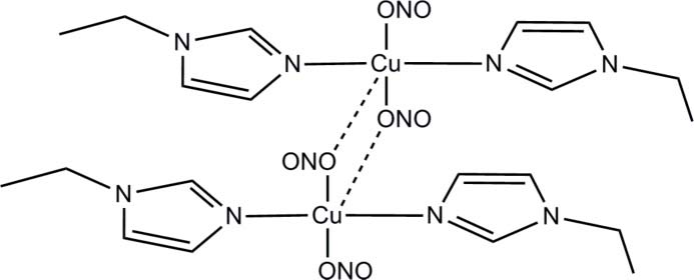


## Experimental

### 

#### Crystal data

[Cu_2_(NO_2_)_4_(C_5_H_8_N_2_)_4_]
*M* *_r_* = 695.64
Tetragonal, 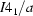

*a* = 28.136 (7) Å
*c* = 7.669 (2) Å
*V* = 6071 (3) Å^3^
*Z* = 8
Mo *K*α radiation
μ = 1.46 mm^−1^
*T* = 293 K
0.30 × 0.25 × 0.20 mm

#### Data collection

Rigaku SCXmini CCD diffractometer
Absorption correction: multi-scan (*CrystalClear*; Rigaku, 2005 ▶) *T*_min_ = 0.651, *T*_max_ = 0.746
31845 measured reflections
3462 independent reflections
3188 reflections with *I* > 2σ(*I*)
*R*_int_ = 0.049

#### Refinement

*R*[*F* ^2^ > 2σ(*F* ^2^)] = 0.049
*wR*(*F* ^2^) = 0.127
*S* = 1.15
3462 reflections
191 parameters
H-atom parameters constrained
Δρ_max_ = 0.82 e Å^−3^
Δρ_min_ = −0.59 e Å^−3^

### 

Data collection: *CrystalClear* (Rigaku, 2005 ▶); cell refinement: *CrystalClear*; data reduction: *CrystalClear*; program(s) used to solve structure: *SHELXS97* (Sheldrick, 2008 ▶); program(s) used to refine structure: *SHELXL97* (Sheldrick, 2008 ▶); molecular graphics: *SHELXTL* (Sheldrick, 2008 ▶); software used to prepare material for publication: *SHELXL97*.

## Supplementary Material

Crystal structure: contains datablock(s) I, global. DOI: 10.1107/S1600536811020745/mw2004sup1.cif

Structure factors: contains datablock(s) I. DOI: 10.1107/S1600536811020745/mw2004Isup2.hkl

Additional supplementary materials:  crystallographic information; 3D view; checkCIF report

## Figures and Tables

**Fig. 1.**
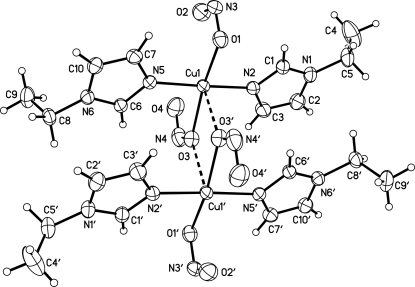
A view of the title compound with the displacement ellipsoids drawn at the 30% probability level.

**Fig. 2.**
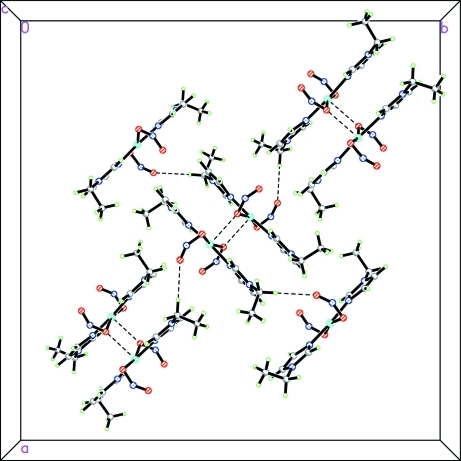
Packing diagram of the title compound. The weak Cu—O interactions and the hydrogen bonds are shown as dashed lines.

## supplementary crystallographic information

### Comment

As part of our ongoing study of potential ferroelectric phase change materials we have determined the structures of several copper complexes and examined the changes in their dielectric constants with temperature. This is the usual method for detecting such behavior. (Fu *et al.*, 2009; Ye *et al.*, 2006; Zhang *et al.*, 2008; Zhang *et al.*, 2010). Unfortunately, the dielectric constant for (I) does not show any behavior indicating the onset of a ferroelectric phase change over the range 80 K to 298 K (m.p.219–229).

As shown in Fig. 1, the Cu ion adopts an elongated square pyramidal geometry with a slight tetrahedral distortion in the basal plane which is primarily associated with the coordination of the nitrite ions (O1—Cu1—O3 = 164.12 (11)°). This displaces O3  from the ideal coordination plane towards the centrosymmetrically-related copper atom (Cu1') resulting in an O3—Cu1' distance of 2.637 (2) Å. While this distance is considerably longer than the in-plane Cu1—O1 and Cu—O3 bond lengths of 2.025 (3) Å and 2.058 (5) Å, respectively, the direction of displacement of O3 and the orientations of the two nitrite ligands which place both O1 and O4 on the opposite side of the coordination plane from Cu1', suggests that there is a weak association of one Cu(NO_2_)_2_(C_5_H_8_N_2_)_2_unit with its centrosymmetrically-related counterpart. A similar weak association has been postulated to occur between two similar centrosymmetrically related Cu(NO_2_)(OC(CH_3_)CHC(CH_3_)N(CH_2_)_2_NH_2_) units (Cu—O = 2.014 (4) Å, Cu'—O = 2.634 (3) Å) (Costes, *et al.* 1995).

### Experimental

An aqueous solution of 1-ethyl imidazole (2.4 g, 25 mmol) and H_2_SO_4_(12.5 mmol) was treated with CuSO_4_ (250 g, 12.5 mmol). After the mixture was stirred for a few minutes, Ba(NO_2_)_2_ (6.18 g, 25 mmol) was added to give a blue solution. Slow evaporation of the solution following removal of the precipitated BaSO_4_ yielded blue crystals after a few days.

### Refinement

All H atoms were placed in geometrically idealized positions and constrained to ride on their parent atoms with C—H = 0.93–0.96 Å, and with *U*_iso_(H) = 1.2 *U*_iso_(C) or 1.5 *U*_iso_(C) for ethy H atoms.

### Crystal data

**Table:**

[Cu_2_(NO_2_)_4_(C_5_H_8_N_2_)_4_]	*D*_x_ = 1.522 Mg m^−^^3^
*M**_r_* = 695.64	Mo *K*α radiation, λ = 0.71073 Å
Tetragonal, *I*4_1_/*a*	Cell parameters from 7423 reflections
Hall symbol: -I 4ad	θ = 2.3–27.5°
*a* = 28.136 (7) Å	µ = 1.46 mm^−^^1^
*c* = 7.669 (2) Å	*T* = 293 K
*V* = 6071 (3) Å^3^	Prism, blue
*Z* = 8	0.30 × 0.25 × 0.20 mm
*F*(000) = 2864	

### Data collection

**Table:**

Rigaku SCXmini CCD diffractometer	3462 independent reflections
Radiation source: fine-focus sealed tube	3188 reflections with *I* > 2σ(*I*)
graphite	*R*_int_ = 0.049
ω scans	θ_max_ = 27.5°, θ_min_ = 2.8°
Absorption correction: multi-scan (*CrystalClear*; Rigaku, 2005)	*h* = −36→35
*T*_min_ = 0.651, *T*_max_ = 0.746	*k* = −36→36
31845 measured reflections	*l* = −9→9

### Refinement

**Table:**

Refinement on *F*^2^	Primary atom site location: structure-invariant direct methods
Least-squares matrix: full	Secondary atom site location: difference Fourier map
*R*[*F*^2^ > 2σ(*F*^2^)] = 0.049	Hydrogen site location: inferred from neighbouring sites
*wR*(*F*^2^) = 0.127	H-atom parameters constrained
*S* = 1.15	*w* = 1/[σ^2^(*F*_o_^2^) + (0.0575*P*)^2^ + 9.3008*P*] where *P* = (*F*_o_^2^ + 2*F*_c_^2^)/3
3462 reflections	(Δ/σ)_max_ = 0.001
191 parameters	Δρ_max_ = 0.82 e Å^−^^3^
0 restraints	Δρ_min_ = −0.59 e Å^−^^3^

### Special details

**Table:**

Geometry. All esds (except the esd in the dihedral angle between two l.s. planes) are estimated using the full covariance matrix. The cell esds are taken into account individually in the estimation of esds in distances, angles and torsion angles; correlations between esds in cell parameters are only used when they are defined by crystal symmetry. An approximate (isotropic) treatment of cell esds is used for estimating esds involving l.s. planes.
Refinement. Refinement of *F*^2^ against ALL reflections. The weighted *R*-factor *wR* and goodness of fit *S* are based on *F*^2^, conventional *R*-factors *R* are based on *F*, with *F* set to zero for negative *F*^2^. The threshold expression of *F*^2^ > σ(*F*^2^) is used only for calculating *R*-factors(gt) *etc*. and is not relevant to the choice of reflections for refinement. *R*-factors based on *F*^2^ are statistically about twice as large as those based on *F*, and *R*- factors based on ALL data will be even larger.

### Fractional atomic coordinates and isotropic or equivalent isotropic displacement parameters (Å^2^)

**Table:**

	*x*	*y*	*z*	*U*_iso_*/*U*_eq_	
Cu1	0.296007 (12)	0.280780 (13)	0.10562 (4)	0.03905 (14)	
O1	0.31265 (8)	0.25812 (8)	−0.1376 (3)	0.0515 (5)	
O2	0.36079 (10)	0.31480 (11)	−0.1169 (4)	0.0703 (7)	
O3	0.26607 (9)	0.28842 (10)	0.3494 (4)	0.0622 (6)	
O4	0.31466 (11)	0.34387 (11)	0.3593 (4)	0.0786 (9)	
N1	0.20821 (9)	0.36827 (10)	−0.1798 (4)	0.0506 (6)	
N2	0.25052 (8)	0.32796 (9)	0.0093 (3)	0.0429 (5)	
N3	0.34610 (10)	0.28278 (12)	−0.2076 (4)	0.0567 (7)	
N4	0.28338 (14)	0.32269 (14)	0.4337 (4)	0.0726 (10)	
N5	0.34476 (8)	0.23769 (8)	0.2053 (3)	0.0385 (5)	
N6	0.38648 (9)	0.19735 (9)	0.3968 (3)	0.0423 (5)	
C1	0.23921 (11)	0.33283 (11)	−0.1568 (4)	0.0463 (7)	
H1	0.2512	0.3140	−0.2462	0.056*	
C2	0.19876 (13)	0.38696 (13)	−0.0200 (5)	0.0615 (9)	
H2	0.1782	0.4120	0.0043	0.074*	
C3	0.22491 (13)	0.36229 (13)	0.0964 (5)	0.0597 (9)	
H3	0.2255	0.3676	0.2161	0.072*	
C4	0.2044 (3)	0.4277 (2)	−0.4124 (8)	0.122 (2)	
H4A	0.1894	0.4348	−0.5217	0.182*	
H4B	0.2382	0.4260	−0.4286	0.182*	
H4C	0.1971	0.4522	−0.3295	0.182*	
C5	0.18697 (14)	0.38208 (15)	−0.3476 (5)	0.0690 (11)	
H5A	0.1901	0.3558	−0.4286	0.083*	
H5B	0.1533	0.3878	−0.3305	0.083*	
C6	0.35428 (10)	0.23166 (10)	0.3735 (4)	0.0405 (6)	
H6	0.3404	0.2490	0.4632	0.049*	
C7	0.37254 (11)	0.20505 (11)	0.1186 (4)	0.0461 (7)	
H7	0.3735	0.2009	−0.0016	0.055*	
C8	0.40610 (12)	0.18264 (12)	0.5666 (4)	0.0511 (7)	
H8A	0.4144	0.1492	0.5617	0.061*	
H8B	0.3820	0.1866	0.6558	0.061*	
C9	0.44914 (14)	0.21084 (15)	0.6157 (6)	0.0697 (11)	
H9A	0.4734	0.2065	0.5291	0.105*	
H9B	0.4607	0.2002	0.7268	0.105*	
H9C	0.4409	0.2439	0.6228	0.105*	
C10	0.39807 (11)	0.18017 (11)	0.2355 (4)	0.0492 (7)	
H10	0.4195	0.1559	0.2113	0.059*	

### Atomic displacement parameters (Å^2^)

**Table:**

	*U*^11^	*U*^22^	*U*^33^	*U*^12^	*U*^13^	*U*^23^
Cu1	0.0390 (2)	0.0460 (2)	0.0321 (2)	0.00571 (13)	0.00052 (13)	0.00114 (13)
O1	0.0565 (13)	0.0524 (12)	0.0458 (12)	0.0022 (10)	−0.0071 (10)	−0.0053 (10)
O2	0.0689 (17)	0.0750 (18)	0.0670 (17)	−0.0149 (14)	−0.0035 (13)	0.0023 (14)
O3	0.0548 (14)	0.0698 (16)	0.0622 (16)	0.0070 (12)	−0.0010 (12)	0.0094 (13)
O4	0.0654 (17)	0.0681 (17)	0.102 (2)	−0.0119 (14)	−0.0124 (16)	0.0019 (16)
N1	0.0453 (14)	0.0531 (15)	0.0533 (16)	0.0069 (11)	−0.0072 (12)	0.0064 (12)
N2	0.0424 (13)	0.0464 (13)	0.0400 (13)	0.0058 (10)	0.0016 (10)	0.0036 (10)
N3	0.0550 (16)	0.077 (2)	0.0380 (14)	0.0110 (14)	0.0067 (12)	0.0059 (14)
N4	0.078 (2)	0.086 (2)	0.0532 (18)	0.031 (2)	−0.0105 (17)	−0.0114 (17)
N5	0.0373 (11)	0.0406 (12)	0.0377 (12)	0.0023 (9)	0.0004 (9)	0.0001 (10)
N6	0.0419 (13)	0.0409 (12)	0.0441 (13)	0.0018 (10)	−0.0018 (10)	0.0061 (10)
C1	0.0435 (15)	0.0509 (17)	0.0446 (16)	0.0051 (13)	−0.0030 (12)	0.0010 (13)
C2	0.058 (2)	0.058 (2)	0.068 (2)	0.0182 (16)	0.0029 (17)	0.0028 (17)
C3	0.065 (2)	0.067 (2)	0.0463 (18)	0.0236 (17)	0.0062 (15)	−0.0030 (16)
C4	0.178 (6)	0.091 (4)	0.096 (4)	−0.014 (4)	−0.050 (4)	0.042 (3)
C5	0.062 (2)	0.079 (3)	0.067 (2)	0.0125 (19)	−0.0202 (18)	0.014 (2)
C6	0.0411 (14)	0.0434 (15)	0.0370 (14)	0.0035 (11)	−0.0009 (11)	0.0003 (11)
C7	0.0506 (17)	0.0440 (15)	0.0437 (16)	0.0043 (13)	0.0045 (13)	−0.0041 (12)
C8	0.0530 (17)	0.0497 (17)	0.0505 (18)	0.0039 (13)	−0.0085 (14)	0.0125 (14)
C9	0.060 (2)	0.067 (2)	0.082 (3)	−0.0045 (17)	−0.028 (2)	0.011 (2)
C10	0.0492 (16)	0.0441 (16)	0.0543 (18)	0.0097 (12)	0.0024 (14)	−0.0011 (14)

### Geometric parameters (Å, °)

**Table:**

Cu1—N5	1.984 (2)	C2—C3	1.350 (5)
Cu1—N2	1.987 (2)	C2—H2	0.9300
Cu1—O1	2.026 (2)	C3—H3	0.9300
Cu1—O3	2.062 (3)	C4—C5	1.460 (7)
O1—N3	1.287 (4)	C4—H4A	0.9600
O2—N3	1.211 (4)	C4—H4B	0.9600
O3—N4	1.259 (4)	C4—H4C	0.9600
O4—N4	1.206 (5)	C5—H5A	0.9705
N1—C1	1.337 (4)	C5—H5B	0.9686
N1—C2	1.360 (5)	C6—H6	0.9300
N1—C5	1.471 (4)	C7—C10	1.345 (4)
N2—C1	1.319 (4)	C7—H7	0.9300
N2—C3	1.378 (4)	C8—C9	1.496 (5)
N5—C6	1.329 (4)	C8—H8A	0.9700
N5—C7	1.377 (4)	C8—H8B	0.9700
N6—C6	1.336 (4)	C9—H9A	0.9600
N6—C10	1.368 (4)	C9—H9B	0.9600
N6—C8	1.474 (4)	C9—H9C	0.9600
C1—H1	0.9300	C10—H10	0.9300
			
N5—Cu1—N2	175.68 (10)	C5—C4—H4B	109.5
N5—Cu1—O1	90.14 (10)	H4A—C4—H4B	109.5
N2—Cu1—O1	90.96 (10)	C5—C4—H4C	109.5
N5—Cu1—O3	89.82 (10)	H4A—C4—H4C	109.5
N2—Cu1—O3	90.25 (10)	H4B—C4—H4C	109.5
O1—Cu1—O3	164.17 (11)	C4—C5—N1	113.2 (4)
N3—O1—Cu1	112.57 (19)	C4—C5—H5A	114.9
N4—O3—Cu1	112.8 (2)	N1—C5—H5A	108.7
C1—N1—C2	107.3 (3)	C4—C5—H5B	103.2
C1—N1—C5	125.3 (3)	N1—C5—H5B	108.8
C2—N1—C5	127.4 (3)	H5A—C5—H5B	107.6
C1—N2—C3	105.6 (3)	N5—C6—N6	111.0 (3)
C1—N2—Cu1	125.7 (2)	N5—C6—H6	124.5
C3—N2—Cu1	128.7 (2)	N6—C6—H6	124.5
O2—N3—O1	114.3 (3)	C10—C7—N5	109.2 (3)
O4—N4—O3	114.7 (3)	C10—C7—H7	125.4
C6—N5—C7	105.6 (2)	N5—C7—H7	125.4
C6—N5—Cu1	126.3 (2)	N6—C8—C9	112.1 (3)
C7—N5—Cu1	127.9 (2)	N6—C8—H8A	109.2
C6—N6—C10	107.2 (2)	C9—C8—H8A	109.2
C6—N6—C8	125.1 (3)	N6—C8—H8B	109.2
C10—N6—C8	127.6 (3)	C9—C8—H8B	109.2
N2—C1—N1	111.3 (3)	H8A—C8—H8B	107.9
N2—C1—H1	124.4	C8—C9—H9A	109.5
N1—C1—H1	124.4	C8—C9—H9B	109.5
C3—C2—N1	106.9 (3)	H9A—C9—H9B	109.5
C3—C2—H2	126.5	C8—C9—H9C	109.5
N1—C2—H2	126.5	H9A—C9—H9C	109.5
C2—C3—N2	108.9 (3)	H9B—C9—H9C	109.5
C2—C3—H3	125.5	C7—C10—N6	107.0 (3)
N2—C3—H3	125.5	C7—C10—H10	126.5
C5—C4—H4A	109.5	N6—C10—H10	126.5
			
N5—Cu1—O1—N3	−89.0 (2)	Cu1—N2—C1—N1	−179.0 (2)
N2—Cu1—O1—N3	86.8 (2)	C2—N1—C1—N2	−0.5 (4)
O3—Cu1—O1—N3	−178.8 (3)	C5—N1—C1—N2	−177.7 (3)
N5—Cu1—O3—N4	90.2 (2)	C1—N1—C2—C3	0.5 (4)
N2—Cu1—O3—N4	−85.4 (2)	C5—N1—C2—C3	177.6 (4)
O1—Cu1—O3—N4	−179.9 (3)	N1—C2—C3—N2	−0.3 (4)
N5—Cu1—N2—C1	109.0 (13)	C1—N2—C3—C2	0.0 (4)
O1—Cu1—N2—C1	4.2 (3)	Cu1—N2—C3—C2	179.3 (2)
O3—Cu1—N2—C1	−160.0 (3)	C1—N1—C5—C4	−109.8 (5)
N5—Cu1—N2—C3	−70.1 (14)	C2—N1—C5—C4	73.6 (6)
O1—Cu1—N2—C3	−174.9 (3)	C7—N5—C6—N6	0.3 (3)
O3—Cu1—N2—C3	20.9 (3)	Cu1—N5—C6—N6	175.07 (18)
Cu1—O1—N3—O2	−0.8 (3)	C10—N6—C6—N5	−0.4 (3)
Cu1—O3—N4—O4	−1.6 (4)	C8—N6—C6—N5	178.0 (3)
N2—Cu1—N5—C6	77.2 (14)	C6—N5—C7—C10	0.0 (3)
O1—Cu1—N5—C6	−178.0 (2)	Cu1—N5—C7—C10	−174.7 (2)
O3—Cu1—N5—C6	−13.8 (3)	C6—N6—C8—C9	−88.7 (4)
N2—Cu1—N5—C7	−109.1 (13)	C10—N6—C8—C9	89.4 (4)
O1—Cu1—N5—C7	−4.3 (2)	N5—C7—C10—N6	−0.3 (4)
O3—Cu1—N5—C7	159.9 (3)	C6—N6—C10—C7	0.4 (3)
C3—N2—C1—N1	0.3 (4)	C8—N6—C10—C7	−178.0 (3)
